# Effect of active TENS versus de-tuned TENS on walking capacity in patients with lumbar spinal stenosis: a randomized controlled trial

**DOI:** 10.1186/s12998-019-0245-z

**Published:** 2019-06-19

**Authors:** Carlo Ammendolia, Pierre Côté, Y. Raja Rampersaud, Danielle Southerst, Michael Schneider, Aksa Ahmed, Claire Bombardier, Gillian Hawker, Brian Budgell

**Affiliations:** 1 Institute of Health Policy, Management and Evaluation, University of Toronto, 60 Murray Street, Rm L2-225, Toronto, Ontario M5T 3L9 Canada; 2 Rebecca MacDonald Centre for Arthritis & Autoimmune Disease, Mount Sinai Hospital, 60 Murray Street, Rm L2-225, Toronto, Ontario M5T 3L9 Canada; 3 Dalla Lana School of Public Health, University of Toronto, Toronto, Canada; 4 UOIT-CMCC Centre for Disability Prevention and Rehabilitation, Faculty of Health Sciences, University of Ontario Institute of Technology, Toronto, Ontario Canada; 5 Department of Orthopedics, Toronto Western Hospital, University Health Network, 399 Bathurst Street, 441, 1 East Wing, Toronto, Ontario M5T 2S8 Canada; 6 Occupational and Industrial Orthopaedic Centre, Department of Orthopaedic Surgery, NYU Langone Health, 63 Downing Street, New York, NY 10014 USA; 7 Department of Physical Therapy, University of Pittsburgh, 100 Technology Drive, Suite 210, Pittsburgh, PA 15219 USA; 8 Department of Medicine, Division of Rheumatology, University of Toronto, 190 Elizabeth Street, Suite RFE 3-805, Toronto, Ontario M5G 2C4 Canada; 9 Department of Medicine, Faculty of Medicine, University of Toronto, P.O. Box 7, 60 Murray Street, Rm L2-008, Toronto, Ontario M5T 3L9 Canada; 10 Canadian Memorial Chiropractic College, 6100 Leslie Street, North York, Ontario M2H 3J1 Canada

**Keywords:** Intermittent claudication, Lumbar spinal stenosis, Transcutaneous electrical nerve stimulation (TENS), Walking, Randomized controlled trial, Non-operative treatment

## Abstract

**Background context:**

Lumbar spinal stenosis (LSS) leads to diminished blood flow to the spinal nerves causing neurogenic claudication and impaired walking ability. Animal studies have demonstrated increased blood flow to the spinal nerves and spinal cord with superficial para-spinal electrical stimulation of the skin.

**Purpose:**

The aim of this study was to assess the effectiveness of active para-spinal transcutaneous electrical nerve stimulation (TENS) compared to de-tuned TENS applied while walking, on improving walking ability in LSS.

**Study design:**

This was a two-arm double-blinded (participant and assessor) randomized controlled trial.

**Patient sample:**

We recruited 104 participants 50 years of age or older with neurogenic claudication, imaging confirmed LSS and limited walking ability.

**Outcome measures:**

The primary measure was walking distance measured by the self-paced walking test (SPWT) and the primary outcome was the difference in proportions among participants in both groups who achieved at least a 30% improvement in walking distance from baseline using relative risk with 95% confidence intervals.

**Methods:**

The active TENS group (*n* = 49) received para-spinal TENS from L3-S1 at a frequency of 65–100 Hz modulated over 3-s intervals with a pulse width of 100–200 usec, and turned on 2 min before the start and maintained during the SPWT. The de-tuned TENS group (*n* = 51) received similarly applied TENS for 30 s followed by ramping down to zero stimulus and turned off before the start and during the SPWT.

Study funded by The Arthritis Society ($365,000 CAN) and salary support for Carlo Ammendolia funded by the Canadian Chiropractic Research Foundation ($500,000 CAN over 5 years).

**Results:**

From August 2014 to January 2016 a total of 640 potential participants were screened for eligibility; 106 were eligible and 104 were randomly allocated to active TENS or de-tuned TENS. Both groups showed significant improvement in walking distance but there was no significant difference between groups. The mean difference between active and de-tuned TENS groups was 46.9 m; 95% CI (− 118.4 to 212.1); *P* = 0.57. A total of 71% (35/49) of active TENS and 74% (38/51) of de-tuned TENS participants achieved at least 30% improvement in walking distance; relative risk (RR), 0.96; 95% CI, (0.7 to 1.2) *P* = 0.77.

**Conclusions:**

Active TENS applied while walking is no better than de-tuned TENS for improving walking ability in patients with degenerative LSS and therefore should not be a recommended treatment in clinical practice.

**Registration:**

ClinicalTrials.gov ID: NCT02592642. Registration October 30, 2015.

## Background

Lumbar spinal stenosis (LSS) causing neurogenic claudication is a leading cause of pain, disability and loss of independence in people over 65 years of age [1]. It is usually caused by age-related osteoarthritic changes of the lumbar spine, leading to narrowing of the spinal canals with associated compression and ischemia of the spinal nerves [2]. LSS is the most common reason for spine surgery in older adults [3]. With an aging population, the prevalence and economic burden of LSS is growing rapidly. The main impairment of LSS is reduced walking ability [4]. Individuals with LSS are more limited in their walking ability compared to individuals with knee or hip osteoarthritis [5]. Moreover, walking impairment in LSS is not likely to improve over time [6].

Neurogenic claudication is the clinical syndrome caused by LSS. It is defined as bilateral or unilateral buttock and lower extremity pain, heaviness, numbness, tingling or weakness, precipitated by standing and walking, and relieved by lumbar flexion [4, 7]. Standing and walking cause further narrowing of the spinal canals which impedes venous return within the spinal canals leading to venous congestion [8–12]. The gradual increase in venous congestion with standing and walking eventually compromises arterial perfusion and leads to hypoxia of the spinal nerves, giving rise to the symptoms of claudication [8]. Sitting and/or stooping forward (lumbar flexion) increases the canal size and relieves venous congestion, thereby restoring blood flow to the spinal nerves [12].

Interventions aimed at reducing venous congestion within the spinal canals and/or increasing blood flow to the spinal nerves while standing and walking may improve symptoms of neurogenic claudication. Recent evidence from animal models demonstrated that innocuous and noxious stimulation to specific dermatomes resulted in a significant increase in blood flow to somatotopically linked spinal cord segments [13–16]. Other animal models have demonstrated an increase in blood flow to the lumbar spinal cord and cauda equina with electrical stimulation of the sciatic nerve [17]. Several human studies have demonstrated significant reduction in laboratory induced ischemic pain in the lower and upper extremities with the application of superficial transcutaneous electrical nerve stimulation (TENS) versus de-tuned TENS [18–22]. Two recent case-controlled studies also demonstrated that 5 min of superficial electrical stimulation of the tibial nerve prior to a walk test significantly improved walking distance in patients with neurogenic claudication [23, 24]. The authors speculated that the nerve stimulation improved blood flow and oxygenation to the spinal nerves of the cauda equina.

There are no randomized controlled trials (RCT) evaluating the effectiveness of TENS applied while walking in patients with neurogenic claudication due to LSS. Therefore, we conducted a randomized controlled trial comparing the effectiveness of active versus de-tuned TENS in improving walking capacity in individuals with neurogenic claudication. We hypothesized that active superficial para-spinal TENS applied while walking would improve walking distance compared to de-tuned superficial para-spinal TENS applied while walking.

## Methods

Trial design and methods were previously published [25]. This and another published study assessing a prototype back belt [26] were nested studies within a larger RCT [27] using the same sample population with a wash out period. Following the baseline assessment all participants were randomized to TENS (*N* = 51) or de-tuned (*N* = 53) and prototype stenosis belt (*N* = 52) or back support (*N* = 52). Half the participants received the TENS or de-tuned intervention first while the other half received the prototype belt or back support first. Following a minimum 2-day washout period, participants initially receiving the TENS or de-tuned TENS received the prototype belt or back support and those who initially received the prototype belt or back support, received the TENS or de-tuned TENS interventions.

The two nested studies had identical objectives and methods (including inclusion and exclusion criteria, randomization, outcomes, sample size calculation and analysis). The only difference was the intervention and controls used. Consequently there is significant overlap between these two nested studies and as well as the published protocol [25].

### Study objective

The objective of this study was to evaluate whether active TENS applied while walking can improve walking distance compared to de-tuned TENS.

### Study design

**Fig. 1:**
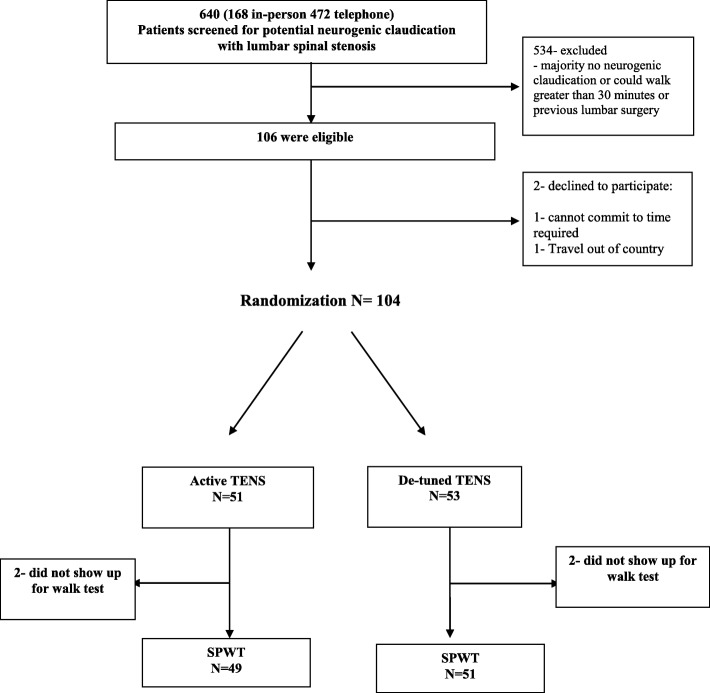
Flow diagram of enrolment and randomization

We conducted a two-arm double-blinded (participant and assessor) single session RCT (Fig. 1), meaning that the intervention and the assessment of walking ability occurred at the same time in a single session.

### Source population

Using an eligibility checklist, interested and potentially eligible participants were referred to the study by medical specialists, family physicians and chiropractors from participating local hospitals and community clinics. Local newspaper advertisements were also used to recruit potential participants. Eligible participants were: 50 years of age or older, had symptoms of neurogenic claudication as defined above for at least 3-months, had imaging-confirmed degenerative spinal canal narrowing, were able to walk without assistance for at least 20 m but could only walk for less than 30 min. Those who had previous surgery for LSS or had other conditions impacting walking ability were excluded from participating in the study (Table 1). A trained study coordinator assessed eligibility, initially screening by phone and then by in-person assessment. At baseline, all eligible and consenting participants completed an intake questionnaire, a short physical performance battery (SPPB) [26] and performed a self-paced walk test (SPWT) [28].

**Table 1:** Inclusion and exclusion criteria

Inclusion criteria	
1. Age greater than or equal to 50 years	
2. Clinical symptoms of back and/or radiating lower limb or buttock pain; fatigue or loss of sensation in the lower limbs aggravated by walking and/or standing and relieved by sitting.	
3. Intermittent or persistent pain without progressive neurological dysfunction	
4. Symptoms and signs for more than 3 months	
5. Imaging-confirmed spinal canal narrowing using MRI, CT scan	
6. Clinical signs and symptoms corresponding to segmental level of narrowing identified by imaging	
7. Patients with degenerative spondylolisthesis are included	
8. Not considered to be a surgical candidate (in the next 12 months) or patient unwilling to have surgery	
9. Able to perform mild-moderate exercise	
10. Able to walk without assistive devices for at least 20 m, but less than 30 min continuously	
11. Able to give written informed consent and complete interviews and questionnaires in English.	
Exclusion criteria	
1. Severe degenerative stenosis with intractable pain and progressive neurological dysfunction	
2. Lumbar spinal stenosis not caused by degeneration	
3. Lumbar herniated disc diagnosed during the last 12 months	
4. Previous back surgery for lumbar spinal stenosis or instability	
5. Underlying spinal disorder such as ankylosing spondylitis, neoplasm, infection or metabolic disease	
6. Intermittent claudication due to vascular disease	
7. Severe osteoarthrosis or arthritis of lower extremities causing limited walking ability	
8. Neurologic disease causing impaired function of the lower limbs, including diabetes	
9. Psychiatric disorders and /or cognitively impaired	

Same Table used in previous published studies [27, 28, 46]

### Protection of human subjects and assessment of safety

#### Ethics, consent and permissions

The hospital institutional review board approved the study (certificate #14–0020-E). There was no commercial sponsorship. No remuneration was provided to participants; travel costs were covered and all interventions were provided free of charge. All participants provided written informed consent. This trial was registered with ClinicalTrials.gov ID: NCT02592642.

#### Randomization

Eligible and consenting participants were randomized to either active para-spinal TENS or de-tuned para-spinal TENS. A biostatistician prepared the randomization sequence using a computerized random number table [NQuery Advisor 7.0]. Sequentially numbered and sealed opaque envelopes containing the sequence were stored in a locked drawer. For each enrolled participant, the study coordinator (not involved in the preparation of the allocation sequence) retrieved and opened the next sequentially numbered envelope and assigned the participant according to the random allocation scheme.

#### Procedures

All participants received their intervention and SPWT within one week of their baseline assessment. The research coordinator applied all the interventions.

#### a) Active Para-spinal TENS

Participants randomized to this subgroup had disposable self-adhesive electrical pads (Blue Sensor P, Ambu A/S, Denmark) applied over the para-spinal musculature from the L3 to S1. The electrodes were connected to a TENS machine [NeuroTrac TENS from Verity Medical Ltd. (U.K.)] that was worn by the participant concealed within a waist pouch. The TENS device was programmed for a frequency of 65–100 Hz modulated over 3-s intervals with a pulse width of 100–200 usec, turned on 2 min before the start and maintained during the SPWT. Current intensity was set to the level of comfort of the patient; approximately 3 mA in pilot experiments, and below the level causing muscle twitch.

#### b) De-tuned Para-spinal TENS

Participants randomized to this subgroup had disposable self-adhesive electrical pads (Blue Sensor P, Ambu A/S, Denmark) applied over the para-spinal musculature from the L3 to S1. The electrodes were connected to a TENS machine [NeuroTrac TENS from Verity Medical Ltd. (U.K.)] that was worn by the participant concealed within a waist pouch. The TENS was programmed according to the protocol of Rakel et al. [29] i.e. the unit provided an active current with a frequency of 65–100 Hz modulated over 3-s intervals with a pulse width of 100–200 usec, turned on 2 min before the start of the SPWT for a duration of 30 s then ramping down to zero stimulus over 15 s and turned off. Participants were led to believe that the unit was still active but providing stimulation below their level of perception.

Participants performed a single SPWT while wearing their assigned device. All SPWTs were performed and recorded by blinded assessors. Blinding was achieved by having participants wear hospital gowns and concealing TENS units within zippered waist pouches. Participants were instructed not to communicate with the assessor beyond answering questions related to the SPWT. A licensed practitioner was nearby during the assessment should the participant experience any discomfort or difficulties related to wearing the device.

### Outcomes

#### Primary measure

##### Objective walking capacity

Walking capacity was assessed using the SPWT. The test required participants to walk on a level surface without support at their own pace until forced to stop due to symptoms of neurogenic claudication or at a time limit of 30 min [30]. Test termination was defined as a complete stop of 3 s. A blinded assessor followed one metre behind the subject, without conversing, with a distance instrument (Lufkin Pro-Series Model PSMW38), and stopwatch. Distance walked and time to test termination was recorded. The SPWT is considered the gold standard with high validity for assessing walking capacity in this population since it directly observes walking ability under conditions representative of a real world setting [30, 31]. It has shown high test-retest reliability (ICC = 0.98) [30].

The primary outcome was the proportion of participants who achieved at least 30% improvement in walking distance (estimated Minimum Clinically Important Difference (MCID)) from baseline assessment. Since there is no validated MCID for the SPWT, a 30% improvement in walking distance was considered appropriate. We also calculated the proportion of participants who achieved at least 50% improvement in walking distance from the baseline assessment.

### Statistical issues

#### Sample size

We estimated the sample size for the primary outcome of objective walking capacity based on an estimate of the difference in the proportion of participants who would achieve the MCID in walking distance from baseline. Since the MCID for the SPWT is unknown we estimated it to be an improvement in walking distance from baseline of 30% or more. We estimated a total of 30% of participants would achieve the estimated MCID in the de-tuned para-spinal TENS group and 60% in the active para-spinal TENS group. Based on an estimate of 30% difference in proportions, a power of 0.8, an alpha of 0.05 and an estimated dropout rate of 20%, a minimum of 52 participants per group was estimated to achieve significance using a two-tailed t-test for two independent proportions [32].

#### Statistical analysis

Baseline status of treatment groups was compared using two-tailed independent samples t tests, Chi squared tests of independence, and Mann-Whitney U tests as indicated. Our analyses were based on the “intention to treat” principle.

We analyzed the primary outcome (SPWT) by calculating the differences in proportions meeting the MCID between the 2 groups using the Pearson Chi Squared test with 95% confidence intervals. We also calculated the relative risk with 95% confidence intervals among participants in both groups who achieved the MCID. To control for potential confounding (sex, education, perceived health status, dominant leg or back pain, and hospital), logistic regression models and generalized estimation equation (GEE) methods were used [33].

#### Adverse events

We measured the presence of adverse events associated with each intervention during the SPWT. We defined adverse events as unintended signs or symptoms arising from the intervention. These included: significant increase in back and/or lower extremity pain, numbness, tingling, tiredness, or claudication symptoms beyond those normally experienced when walking. We computed the incidence (95% CI) of each adverse event listed above. The total number of participants was used as the denominator.

## Results

From August 2014 to January 2016 a total of 640 potential participants were screened for eligibility; 106 were eligible and 104 were randomly allocated to active TENS or de-tuned TENS (Fig. 1). The two groups were similar at baseline (Table 2). The mean age of the study sample was 70·6 years, 57% were female, 84% had leg symptoms for more than 12-months and the mean maximum distance walked without rest at baseline was 329.2 m.

**Table 2:** Baseline characteristics of the study participants*

Variable	TENS (*N* = 51)	De-tuned TENS (*N* = 53)
Age - years	69.4 ± 9.2	71.7 ± 8.2
Sex- no. (%)		
Male	18 (35)	27 (51)
Female	33 (65)	26 (49)
Marital status- no. (%)		
Single, never married	4 (8)	4 (8)
Married	28 (55)	31 (58)
Common-law	2 (4)	6 (11)
Divorced	9 (18)	6 (11)
Widowed	7 (14)	6 (11)
Separated	1 (2)	0 (0)
Expectations- no. (%)		
Get better soon Get better slowly	12 (24) 15 (29)	8 (15) 21 (40)
Never get better	8 (16)	6 (11)
Don’t know	16 (31)	18 (34)
Global Health rating†	68.3 ± 14.6	68.7 ± 15.5
Comorbidities- no. (%)^		
Yes	38 (75)	37 (70)
No	12 (24)	16 (30)
Unknown	1 (2)	0 (0)
Duration of back pain- no. (%)		
< 3 months	0 (0)	1 (2)
3 to 12 months	10 (20)	4 (8)
> 12 months	41 (80)	48 (91)
Duration of leg pain- no. (%)		
3 to 12 months	11 (22)	6 (11)
> 12 months	40 (78)	47 (89)
Dominant pain- no. (%)		
Leg	30 (59)	36 (68)
Back	12 (24)	10 (19)
Equal	9 (18)	7 (13)
Zurich Claudication Questionnaire (ZCQ)		
ZCQ Function score‡	0.6 ± 0.1	0.6 ± 0.1
ZCQ Symptoms score¶	0.6 ± 0.1	0.6 ± 0.1
Oswestry Disability Index (ODI)║	0.4 ± 0.1	0.4 ± 0.1
ODI walk- no. (%)^^		
No limitations	0 (0)	0 (0)
2 km	3 (6)	6 (11)
1 km	10 (20)	18 (34)
500 m	37 (73)	28 (53)
Gait aid	1 (2)	1 (2)
Bedridden	0 (0)	0 (0)
Numeric Rating Scale (NRS)		
NRS-Back pain‡‡	5.9 ± 2.7	5.0 ± 2.6
NRS-Leg pain¶¶	7.4 ± 2.0	6.7 ± 2.2
Falls Efficacy Scale§§	31.3 ± 21.4	30.2 ± 20.1
SF36 Subscales††		
SF36-PF	35.2 ± 19.7	40.0 ± 23.3
SF36-MH	68.4 ± 18.6	73.0 ± 18.8
SF36-BP	37.6 ± 15.5	43.8 ± 19.1
Center for Epidemiological Studies-Depression	12.3 ± 9.6	11.0 ± 9.9
(CES-D) scale***		
Self-Paced Walk Test (SPWT)- meters‡‡‡	353.2 ± 381.1	305.1 ± 301.2

*Similar Table with different data published previously [26]

^*^Plus-minus values are means ±SD

^*^There were no significant between group differences in any of the remaining baseline characteristics

†Global health rating scores range from 0 to 100, with higher scores indicating better health

^Comorbidities include: problems with other muscle, bone or joint conditions, allergies, breathing, hypertension, heart and circulation, digestive system, diabetes, kidney and genitourinary, neurological, headaches, mental or emotional and cancer

‡ZCQ Function scores range from 0.25 to 1.0, with lower scores indicating less severity (score range converted from 1 to 4)

¶ZCQ Symptom scores range from 0.20 to 1.0, with lower scores indicating less severity (score range converted from 1 to 5)

║ODI scores range from 0 to 1.0, with lower scores indicating less disability

^^ODI walk allows for 6 possible responses on walking ability; no limitations, 2 km, 1 km, 500 m, gait aid, bedridden

‡‡NRS-Back Pain scores range from 0 to 10, with 0 indicating no pain and 10 indicating “pain as bad as you can imagine”

¶¶NRS-Leg Pain scores range from 0 to 10, with 0 indicating no pain and 10 indicating “pain as bad as you can imagine”

§§Falls Efficacy Scale scores range from 10 to 100, with lower scores indicating less severity

††SF36 Subscales range from 0 to 100, with lower scores indicating poorer health. *PF* Physical Function, *MH* Mental Health, *BP* Bodily Pain

***CES-D scores range from 0 to 60, with lower scores indicating less depressive symptomatology

‡‡‡SPWT measures objective walking distance in meters without stopping due to neurogenic claudication symptoms

With active TENS and de-tuned TENS applied while walking, both groups showed significant improvement in walking distance during the SPWT. The active TENs group walked an additional 210.1 m compared to an additional 163.3 m walked by the de-tuned TENS group. However, the between-group difference was not statistically significant, with a mean difference of 46.9 m; 95% confidence intervals (CI), − 118.4 to 212.1; *P* = 0.57 (Table 3).

**Table 3:** Intention to treat analysis comparing TENS and de-tuned TENS while Walking*

Outcome	Baseline	Active TENS	De-tuned TENS	Treatment effect	*P*-value
		Mean difference from baseline with 95% CI		Adjusted Treatment effect with 95% CI	*P* value
Primary Outcomes					
*No. of Participants*	104	49	51		
SPWT Distance meters		210.1 (70.0 to 350.2)	163.3 (72.5 to 254.1)	46.9 (−118.4 to 212.1)	0.57
		Percentage with 95% CI		Relative Risk with 95% CI	
*>* *30% improvement in SPWT - % [N]*		71 (57, 82) [35/49]	74 (60, 84) [38/51]	0.96 (0.7 to 1.2)	0.77
Secondary Outcome					
*>* *50% improvement in SPWT- % [N]*		69 (55, 80) [34/49]	69 (56, 80) [35/51]	0.99 (0.8 to 1.3)	0.94

*Similar Table with different data published previously [26]

A total of 71% (35/49) of active TENS participants demonstrated at least 30% improvement in walking distance compared to 74% (38/51) of de-tuned TENS participants, Relative Risk, RR; 0.96; 95% CI, 0.7 to 1.2; *P* = 0.77 (Table 3).

A total of 69% (34/49) of active TENS and 69% (35/51) of de-tuned TENS participants demonstrated at least 50% improvement in walking distance, relative risk, RR; 0.99; 95% CI, 0.8 to 1.3; *P* = 0.94 (Table 3).

There were no reported significant adverse events in either group.

## Discussion

In this participant and assessor blinded RCT, we found the application of active TENS to be no better than de-tuned TENS in improving walking ability among patients with neurogenic claudication. However, both the active TENS and de-tuned TENS participants demonstrated significant and clinically important improvement in walking ability. We also found a large proportion of participants in both groups who demonstrated at least 30% improvement in their walking ability, but again with no statistically significant between-group differences.

The similarity in walking improvement of the two treatments may be due to a number of factors. In animal studies the increase blood flow to the spinal cord and cauda equina with para-spinal superficial electrical stimulation was determined by the intensity of the electrical stimulus [13, 17]. Therefore, it is possible that the stimulus intensity used in the active TENS group was not sufficient to produce a clinical response discernable from that of de-tuned TENS.

Furthermore, as innocuous mechanical stimulation of the skin has been shown to produce augmented spinal cord blood flow in animal studies, the presence of the adhesive electrodes alone may have been sufficient to obscure any effects attributable to electrical stimulation [14, 15]. Moreover, the initial TENS stimulation for 30 s followed by the ramping down over 15 s in de-tuned TENS may have had a physiological effect on blood flow to the spinal nerves that was sustained during the SPWT [13].

In addition to the potential physiological improvement in blood flow through neuro-stimulation (noxious and innocuous), improved walking ability seen in both groups may have been partially or totally due to placebo effects. Placebo responses in trials for low back pain can be large and clinically significant even in open-label placebo trials [34]. The placebo effects are thought to be due to the psychosocial effects of the therapeutic encounter, including its interactions, rituals and symbols [35]. The placebo effect may alter patient beliefs and provide hope that the treatment might be helpful. Patients with neurogenic claudication due to LSS have high levels of anxiety, depression and hopelessness [36]. Engendering hope when participants feel hopeless about their condition can be therapeutic and patient expectations may produce independent and powerful placebo analgesic effects [37, 38].

This is the first randomized clinical trial assessing TENS while walking in LSS. Two recent human studies showed improved walking ability in patients with neurogenic claudication with stimulation of the tibial nerve prior to walking, [23, 24]. However, these studies were of low methodological quality.

There have been a number of published RCTs assessing various non-operative treatments for LSS. Systematic reviews of these RCTs concluded that current trials were of low methodological quality; therefore, no conclusions could be made about the effectiveness of non-operative interventions including their benefit on walking ability [39–43].

The lack of significant improvement with active TENS compared to de-tuned TENS suggests that active TENS should not be recommended as a treatment option for patients with neurogenic claudication. However the large treatment effects seen in both groups warrants further study.

The strengths of this study were the use of a randomised controlled design where participants and assessors were blinded, a very low dropout rate and the use of a valid and objective primary outcome measure that is highly meaningful to patients with LSS [36].

This study was a nested study with another study comparing a prototype stenosis belt to a back support and therefore these interventions may have had a carry-over effect that may have influenced the results. However, each of the interventions (TENS or de-tuned TENS and prototype stenosis belt or back support) was assessed using a single walk test lasting a mean of approximately 8 min. The short mean duration of the interventions and the minimum 2-day wash over period would make any potential carry-over effects unlikely.

Further studies using different stimulation parameters are needed to determine whether alternative parameters could produce clinically meaningful and statistically significant benefits. Other comparators may need to be considered other than the de-tuned TENS used in this study, since it may have produced unexpected physiological effects. Adding a third arm to this trial, with an inactive component may have been useful to control for potential non-specific effects. The sustainability of the treatment effects seen in this study requires further investigation using longer-term follow-up. We did not quantify the severity of MRI findings among participants in this study. However, MRI findings in LSS generally have limited correlation with patient symptoms or functional abilities [44, 45]. Moreover, the population of interest in this study was individuals with neurogenic claudication, which by definition is a clinical diagnosis and MRI findings are not required. Finally, more high quality RCTs are needed to assess non-operative treatment options both new and existing for LSS.

## Conclusions

Active TENS was found to be no better than de-tuned TENS and should not be a recommended treatment option for patients with limited walking ability due to neurogenic claudication. The large treatment effects seen in both groups warrant further study.

## Abbreviations

CI: confidence intervals
GEE: generalized estimation equation
LSS: Lumbar spinal stenosis
MCID: minimum clinically important difference
RCT: randomized controlled trial
SPPB: short physical performance battery
SPWT: self-paced walk test
TENS: transcutaneous electrical nerve stimulation
